# The joint effect of vitamin-D status and tobacco exposure on overweight and obesity in children

**DOI:** 10.1017/S0007114524002071

**Published:** 2024-11-28

**Authors:** Yanyan Lin, Gulan Zeng, Yanyan Sun

**Affiliations:** 1 Department of Child Health, Department of Pediatrics, Women and Children’s Hospital, School of Medicine, Xiamen University, Xiamen, Fujian 361102, People’s Republic of China; 2 Department of Child Health Care, Fujian Maternity and Child Health Hospital, Fuzhou, Fujian 350001, People’s Republic of China; 3 Department of Traditional Chinese Medicine, Children’s Hospital of Fudan University, Shanghai 201102, People’s Republic of China

**Keywords:** Vitamin-D status, Tobacco exposure, Overweight, Obesity, Children

## Abstract

This study aimed to explore the combined effects of serum vitamin-D level and tobacco exposure on the risk of overweight and obesity in children. This cross-sectional study analysed the data of 11 636 children aged 2–17 years from the National Health and Nutrition Examination Surveys database between 2007 and 2018. Univariable and multivariate weighted logistic regression models were used to analyse the associations of serum vitamin-D or cotinine levels with overweight and obesity in children as well as the combined effects of serum vitamin-D and cotinine on the risk of overweight and obesity in children. Subgroup analysis was performed in terms of gender, age, race and household smokers. OR with corresponding 95 % CI was presented. The elevated risk of overweight and obesity in children was found in those with serum vitamin-D < 20 ng/ml (OR = 1·44, 95 % CI: 1·29, 1·61). Also, the odds of overweight and obesity in children was 1·14 (OR = 1·14, 95 % CI: 1·01, 1·29) in children with cotinine ≥ 0·05 ng/ml. Relative to participants with serum vitamin-D ≥ 20 ng/ml and cotinine < 0·05 ng/ml, increased risk of overweight and obesity was identified in those with serum vitamin-D < 20 ng/ml and cotinine < 0·05 ng/ml (OR = 1·45, 95 % CI: 1·26, 1·68) and serum vitamin-D < 20 ng/ml and cotinine ≥ 0·05 ng/ml (OR = 1·62, 95 % CI: 1·38, 1·91). Serum vitamin-D and cotinine exposure had combined effects on the risk of overweight and obesity in children.

Childhood obesity is one of the most threatening and alarming public health problems in the world^(1)^. The estimated prevalence of overweight or obesity among children under the age of 5 was approximately 39 million in 2020 and for children aged 5–19 years, it was around 150 million. These numbers are projected to increase to approximately 40 million and 254 million, respectively, by the year 2030^(2)^. Childhood obesity has the potential to persist into adulthood and leads to adverse cardiovascular outcomes or other obesity-related diseases^(3,4)^. Therefore, to identify modifiable risk factors for overweight and obesity in children is essential to implement corresponding interventions to reduce the burden of disease.

The aetiology of obesity is complex, and inflammation and oxidative stress are considered to be the key mechanisms of obesity^(5)^. Environmental tobacco exposure is a major health problem for children, and exposure to tobacco smoke was associated with increased inflammatory responses, oxidative stress and endocrine disorders, which may promote the development of obesity^(6,7)^. Tobacco exposure mainly includes active smoking and second-hand smoke exposure. Cotinine, a direct metabolite of nicotine in tobacco, is a specific and sensitive marker of recent tobacco smoke exposure^(8)^. Previous evidence indicated that higher levels of cotinine were associated with increased risk of childhood obesity in both active and passive smokers^(9)^.

Vitamin-D deficiency is one of the most common nutritional problems, which is considered an anti-inflammatory nutrient, and vitamin-D deficiency may affect the risk of obesity through chronic low-grade inflammation^(10)^. Epidemiological investigations revealed that vitamin-D levels were lower in smokers than in non-smokers, and a positive correlation between tobacco exposure and vitamin-D deficiency was identified^(11–13)^. Animal experiments found that vitamin-D deficiency exacerbated the elevation of proinflammatory cytokines and chemokines caused by tobacco smoke^(14)^. In addition, vitamin-D and tobacco exposure were found to have combined effects on many diseases such as hypertension in adults^(15–18)^. Thus, we suspected that vitamin-D and tobacco exposure might have combined effects on the risk of overweight and obesity in children.

This study aimed to explore the combined effects of serum vitamin-D level and tobacco exposure on the risk of overweight and obesity in children based on data from the National Health and Nutrition Examination Surveys (NHANES). The analysis was also stratified by gender, age, race and household smokers.

## Methods

### Study design and population

This cross-sectional study involved in the data of 19 155 children aged 2–17 years from the NHANES database from 2007 to 2018. The NHANES was a nationally representative survey of nutrition and health condition in the USA (https://wwwn.cdc.gov/nchs/nhanes/Default.aspx). The survey integrated health interviews conducted in respondents’ residences with health measurements carried out at mobile exam centres (MEC). The examination components encompassed medical, dental and physiological measurements, as well as laboratory tests supervised by trained medical personnel^(19)^. In our study, individuals aged 2–18 years with the assessment of height and weight and complete data of serum vitamin-D and cotinine were identified. Participants without complete information of key covariates were excluded. This study has been exempted from an ethical review in our study because this study used the data from public database. The NHANES survey got the NCHS Ethics Review Board (ERB) approval (2007–2008 (Continuation of Protocol #2005-06), 2009–2010 (Continuation of Protocol #2005-06), 2011–2012 (Protocol #2011-17), 2013–2014 (Continuation of Protocol #2011-17), 2015–2016 (Continuation of Protocol #2011-17, and 2017–2018 (Continuation of Protocol #2011-17 & Protocol #2018-01)).

### Potential covariates and definitions

Age (years), gender (boys or girls), race (White, Black or other), PIR (< 1·3, ≥ 1·3 or unknown), education level (below high school, high school or above high school), birth weight (< 5·5 pounds, ≥ 5·5 pounds or unknown), physical activity (not ideal physical activity, ideal physical activity or unknown), sedentary time (< 5 h, or ≥ 5 h), maternal smoking during pregnancy or not, household smokers (nobody or ≥ 1), energy (kcal), fat (gm), protein (gm) and carbohydrate/fibre and dietary vitamin-D (mcg) were potential covariates analysed in our study.

Physical activity was converted into energy consumption, which was calculated according to the response in the questionnaire. Energy consumption (metabolic equivalent of task × min) = metabolic equivalent of task (metabolic equivalent of task) × the duration of the corresponding activity (min). Ideal physical activity was defined as ≥ 180 metabolic equivalent of task × min/day or ≥ 60 min/day (for children aged 2–11 years old), otherwise it was not ideal physical activity. Through the individual’s daily hours of television, video or computer usage based on in-person interview data and categorised into three groups: < 3 h, 3–6 h and ≥ 6 h^(20)^.

### Main variables

Serum vitamin-D and cotinine levels were main variables.

Serum vitamin-D was calculated as follows:

25 (OH) D2:1 nmol/l = 0·41266 ng/ml.

25 (OH) D3:1 nmol/l = 0·40066 ng/ml.

Vitamin-D (ng/ml) = 25 (OH) D2 × 0·41266 × 0·40066 + 25 (OH) D3. Serum vitamin-D status was classified as 20 ng/ml for adequate and < 20 ng/ml for insufficient^(21,22)^.

The serum 25(OH)D concentrations were measured at the National Center for Environmental Health, CDC, Atlanta, GA using the DiaSorin RIA kit (Stillwater MN). The manufacturer reformulated the DiaSorin assay kit used to measure 25(OH)D in NHANES III in 1998 (after completion of NHANES III analyses), incorporating: (1) an improved binding antibody and (2) a modified washing solution to reduce non-specific binding. To evaluate the impact of these changes on observed trends in population serum 25(OH)D levels, a subset of banked serum samples from NHANES III was reanalysed by the CDC laboratory using the reformulated version of the RIA. The NHANES III results obtained with the reformulated assay were compared against those obtained with the original assay for these reanalysed specimens (https://wwwn.cdc.gov/Nchs/Nhanes/vitamind/analyticalnote.aspx?b=2007&e=2008&d=VID_E&x=htm).

Cotinine was identified through the serum levels of cotinine, which was grouped into cotinine ≥ 0·05 ng/ml (the presence of tobacco exposure) and cotinine < 0·05 ng/ml (the absence of tobacco exposure)^(23)^. Serum cotinine is quantified using an isotope dilution-high performance liquid chromatography/atmospheric pressure chemical ionisation tandem mass spectrometry (ID HPLC-APCI MS/MS) method. Briefly, the serum sample is spiked with methyl-D3 cotinine as an internal standard and subjected to a basified solid-phase extraction column for equilibration. Cotinine is extracted from the column using methylene chloride, and the organic extract is concentrated before being injected onto a short C18 HPLC column. The eluate from these injections undergoes APCI-MS/MS monitoring, with quantitation of the m/z 80 daughter ion from the m/z 177 quasi-molecular ion along with additional ions for internal and external standards and confirmation purposes. Cotinine concentrations are determined by comparing native to labelled cotinine ratios in samples against a standard curve (–https://wwwn.cdc.gov/Nchs/Nhanes/2007–2008/COTNAL_E.htm).

### Outcome

Overweight and obesity in children was the outcome, which was evaluated by BMI. For children, BMI was converted to a BMI z-score accounting for age and sex using the recommended CDC percentiles. A BMI z-score of ≥ 85th percentile and < 95th percentile indicates overweight status, and a BMI z-score of ≥ 95th percentile indicates obesity^(24)^ (About Child & Teen BMI | Healthy Weight, Nutrition, and Physical Activity | CDC).

### Statistical analysis

Measurement data were described as mean and se, and weighted *t* test was used for comparison between groups. Enumeration data were described as number and percentage of cases (*n* (%)), and χ^2^ test was used for comparison between groups. SDMVPSU, SDMVSTRA and WTMEC2YR were weight used in this study. Missing values are shown in online Supplementary Table 1. The mean matching prediction method was used to fill in the missing variables, and sensitivity analysis was performed before and after missing values manipulation. The results delineated that no significant difference was found between the data of before and after missing values manipulation (online Supplementary Table 2). Potential covariates were identified via univariable weighted logistic regression model. Univariable and multivariate weighted logistic regression models were used to analyse the associations of serum vitamin-D or cotinine levels with overweight and obesity in children as well as the combined effects of serum Vitamin-D and cotinine on the risk of overweight and obesity in children. Subgroup analysis was performed in terms of gender, age, race and household smokers. The relative excess risk due to interaction, the attributable proportion due to interaction (AP) and the synergy index (S) were applied to evaluate the interaction of serum vitamin-D and cotinine on the risk of overweight and obesity in children. OR with corresponding 95 % CI were presented. SAS 9.4 was applied for statistical analysis. *P* < 0·05 was considered statistical significance.

## Results

### The characteristics of children with underweight and healthy weight or overweight and obesity

**Fig. 1.**
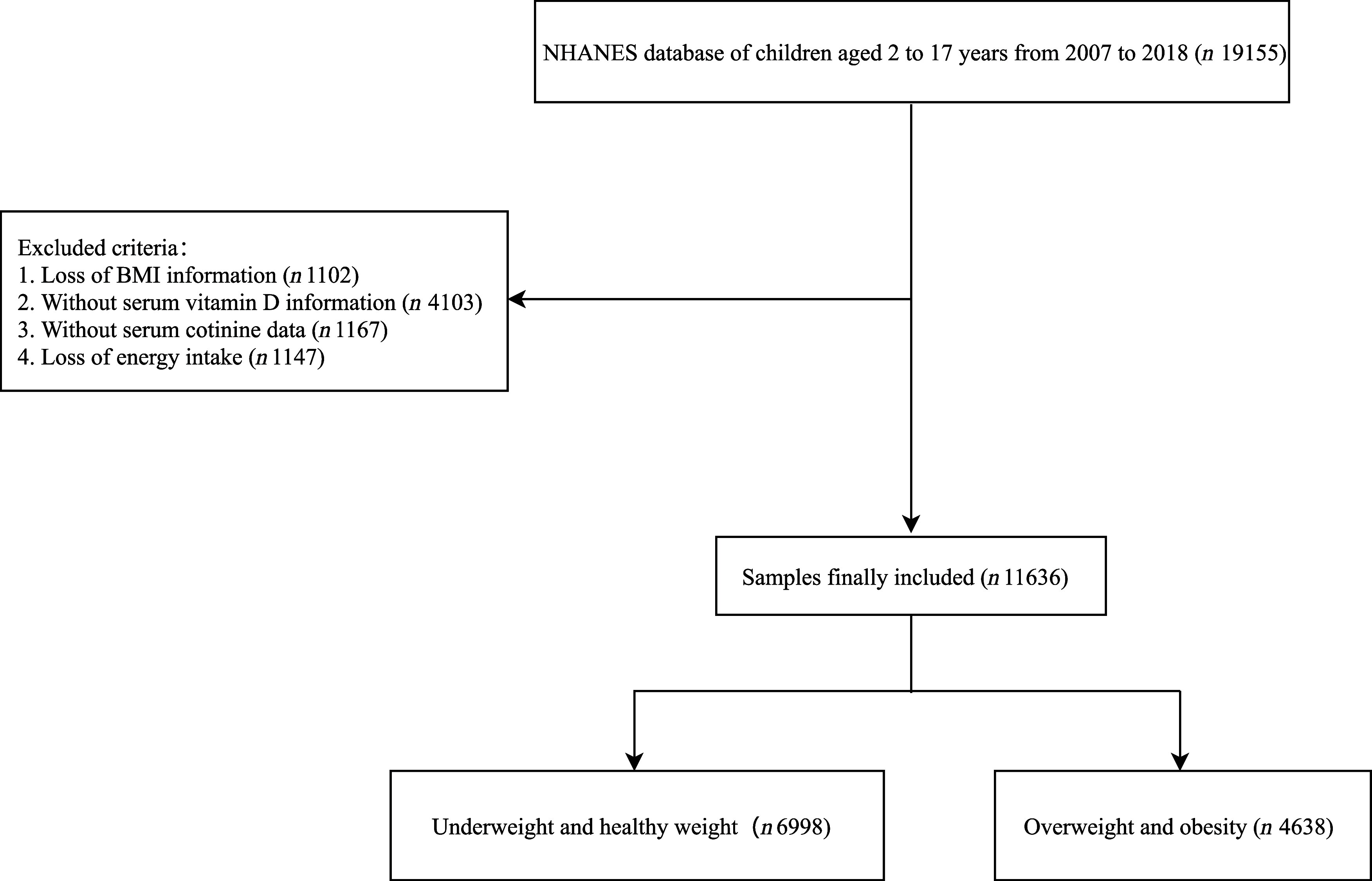
The flow chart of screening process of subjects. NHANES, the National Health and Nutrition Examination Surveys.

In total, the data of 19 155 children aged 2–18 years old were identified from NHANES database. BMI is crucial for defining overweight and obesity in children. Vitamin-D and cotinine were main variables in this study, missing these data could not be analysed. Among them, those without information on BMI assessment (*n* 1102), serum vitamin-D (*n* 4103) and cotinine (*n* 1167) were identified. Energy intake might affect BMI status of children. Also, individuals without energy intake data were excluded (*n* 1147). Finally, 11 636 children were included. All the participants were divided into underweight and healthy weight group (*n* 6998), and overweight and obesity group (*n* 4638). The screening process of all participants was presented in Fig. 1.

As observed in Table 1 and Table 2, the mean serum vitamin-D level in the overweight and obesity group was lower than the underweight and healthy weight group (25·14 ng/ml *v*. 28·23 ng/ml). The percentage of participants with serum vitamin-D ≥ 20 ng/ml in the overweight and obesity group was lower than the underweight and healthy weight group (75·81 % *v*. 84·59 %). The percentage of children with cotinine level ≥ 0·05 ng/ml in the overweight and obesity group was higher than the underweight and healthy weight group (44·25 % *v*. 39·14 %). More information on the characteristics of participants are depicted in Table 1 and Table 2.

**Table 1.** The characteristics of participants with different BMI (Mean values with their standard errors; numbers and percentages)

			BMI (Z-score)					
	Total (*n* 11 636)		Underweight and Healthy Weight (*n* 6998)		Overweight and obesity (*n* 4638)			
Variables	Mean	se	Mean	se	Mean	se	Statistics	*P*
Age (years)	10·81	0·06	10·58·	0·08	11·18	0·08	t = –5·53	< 0·001
	*n*	%	*n*	%	*n*	%		
Gender							χ^2^ = 0·372	0·542
Male	5978	51·45	3651	51·73	2327	50·98		
Female	5658	48·55	3347	48·27	2311	49·02		
Race							χ^2^ = 66·212	< 0·001
White	3308	54·07	2155	57·27	1153	48·87		
Black	2778	13·63	1635	12·75	1143	15·06		
Other	5550	32·30	3208	29·98	2342	36·07		
PIR							χ^2^ = 31·814	< 0·001
< 1·3	4866	31·09	2819	28·95	2047	34·57		
≥ 1·3	5916	63·13	3684	65·55	2232	59·22		
Unknown	854	5·77	495	5·50	359	6·21		
Education level							χ^2^ = 63·845	< 0·001
Below high school	3121	20·43	1742	18·38	1379	23·76		
High school	3254	27·16	1884	25·88	1370	29·22		
Above high school	5261	52·41	3372	55·74	1889	47·02		
Birth weight (pounds)							χ^2^ = 4·247	0·120
< 5·5	1269	9·49	842	10·11	427	8·49		
≥ 5·5	8728	74·00	5192	73·49	3536	74·83		
Unknown	1639	16·51	964	16·40	675	16·68		
Physical activity							χ^2^ = 18·331	< 0·001
Not ideal physical activity	2908	23·41	1658	21·79	1250	26·04		
Ideal physical activity	7857	67·95	4851	69·83	3006	64·90		
Unknown	871	8·64	489	8·38	382	9·06		
Sedentary time (hours)							χ^2^ = 23·928	< 0·001
< 5	7467	63·36	4639	65·56	2828	59·81		
≥ 5	4169	36·64	2359	34·44	1810	40·19		
Maternal smoking during pregnancy							χ^2^ = 13·992	< 0·001
No	8633	71·17	5220	72·27	3413	69·38		
Yes	1232	11·42	713	10·21	519	13·37		
Unknown	1771	17·41	1065	17·52	706	17·24		
Household smokers							χ^2^ = 18·417	< 0·001
Nobody	10 168	88·39	6157	89·45	4011	86·67		
≥ 1	1468	11·61	841	10·55	627	13·33		
	Mean	se	Mean	se	Mean	se		
Energy (kcal)	1968·40	10·35	1978·99	13·22	1951·22	16·39	t = 1·33	0·188
Fat (gm)	74·82	0·57	74·87	0·66	74·74	0·92	t = 0·12	0·904
Protein (gm)	69·51	0·51	69·12	0·64	70·15	0·73	t = –1·14	0·257
Carbohydrate (gm)	259·57	1·33	262·69	1·80	254·49	2·00	t = 2·99	0·004
Fibre (gm)	14·02	0·11	14·11	0·13	13·86	0·15	t = 1·48	0·141
Carbohydrate/Fibre	21·62	0·19	21·62	0·22	21·63	0·33	t = –0·05	0·960

PIR, poverty to income ratio.

**Table 2.** The serum Vitamin-D and cotinine status of participants with different BMI (Mean values with their standard errors; numbers and percentages)

			BMI (Z-score)					
	Total (*n* 11 636)		Underweight and Healthy Weight (*n* 6998)		Overweight and obesity (*n* 4638)			
Variables	Mean	se	Mean	se	Mean	se	Statistics	*P*
Dietary Vitamin-D (mcg)	7·67	0·28	7·99	0·39	7·16	0·40	t = 1·46	0·148
Serum Vitamin-D (ng/ml)	27·05	0·29	28·23	0·31	25·14	0·29	t = 14·06	< 0·001
	*n*	%	*n*	%	*n*	%		
Serum Vitamin-D (ng/ml)							χ^2^ = 131·205	< 0·001
< 20	2998	18·76	1544	15·41	1454	24·19		
≥ 20	8638	81·24	5454	84·59	3184	75·81		

	Mean	se	Mean	se	Mean	se		
Cotinine (ng/ml)	3·54	0·40	3·68	0·55	3·31	0·53	t = 0·49	0·622
	*n*	%	*n*	%	*n*	%		
Cotinine (ng/ml)							χ^2^ = 20·956	< 0·001
< 0·05	6527	58·91	4009	60·86	2518	55·75		
≥ 0·05	5109	41·09	2989	39·14	2120	44·25		
Serum Vitamin-D: Cotinine							χ^2^ = 137·552	< 0·001
≥ 20: < 0·05	4909	48·66	3159	52·23	1750	42·88		
≥ 20: ≥ 0·05	3729	32·58	2295	32·36	1434	32·93		
< 20: < 0·05	1618	10·25	850	8·63	768	12·87		
< 20: ≥ 0·05	1380	8·51	694	6·78	686	11·32		

PIR: poverty to income ratio.

### Combined effects of serum vitamin-D and cotinine on the risk of overweight and obesity in children

The potential covariates are shown in online Supplementary Table 3, which indicated that age, race, PIR, education, birth weight, physical activity, sedentary time, maternal smoking during pregnancy and household smokers were covariates associated with overweight and obesity in children.

**Fig. 2.**
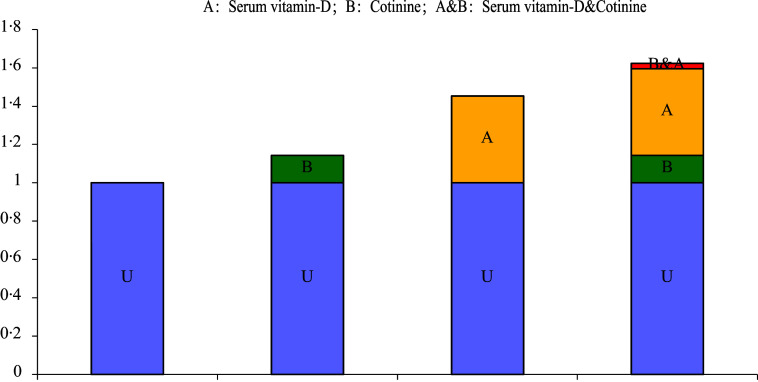
The combined effects of serum Vitamin-D and cotinine on the risk of overweight and obesity in children.

In the univariable analysis, serum vitamin-D < 20 ng/ml was associated with increased risk of overweight and obesity in children compared with those with serum vitamin-D ≥ 20 ng/ml (OR = 1·75, 95 % CI: 1·58, 1·95). After adjusting for relevant covariates, the association between serum vitamin-D < 20 ng/ml and elevated risk of overweight and obesity in children was still significant (OR = 1·44, 95 % CI: 1·29, 1·61). The crude model revealed that compared with children with cotinine < 0·05 ng/ml, subjects with cotinine ≥ 0·05 ng/ml was a risk factor for overweight and obesity (OR = 1·23, 95 % CI: 1·12, 1·35). In the adjusted model, the odds of overweight and obesity in children was 1·14 (OR = 1·14, 95 % CI: 1·01, 1·29) in children with cotinine ≥ 0·05 ng/ml. Relative to participants with serum vitamin-D ≥ 20 ng/ml and cotinine < 0·05 ng/ml, increased risk of overweight and obesity was identified in those with serum vitamin-D < 20 ng/ml and cotinine < 0·05 ng/ml (OR = 1·45, 95 % CI: 1·26, 1·68) and serum vitamin-D < 20 ng/ml and cotinine ≥ 0·05 ng/ml (OR = 1·62, 95 % CI: 1·38, 1·91) (Table 3). The combined effects of serum vitamin-D and cotinine on the risk of overweight and obesity in children are exhibited in Fig. 2. No significant interaction between serum vitamin-D and cotinine on the risk of overweight and obesity in children was identified (relative excess risk due to interaction = 0·028, 95 % CI: −0·256, 0·312), AP = 0·017, 95 % CI: −0·157, 0·191), S = 1·047, 95 % CI: 0·654, 1·675)) (online Supplementary Table 4).

**Table 3.** Combined effects of serum vitamin-D and cotinine on the risk of overweight and obesity in children (Odds ratios and 95 % confidence intervals)

	Model 1			Model 2			
Variables	OR	95 % CI	*P*	OR	95 % CI	*P*	*P*_of trend_
Serum Vitamin-D							–
≥ 20 ng/ml	Ref			Ref			
< 20 ng/ml	1·75	1·58, 1·95	< 0·001	1·44	1·29, 1·61	< 0·001	
Cotinine							
< 0·05 ng/ml	Ref			Ref			
≥ 0·05 ng/ml	1·23	1·12, 1·35	< 0·001	1·14	1·01, 1·29	0·034	
Serum Vitamin-D: Cotinine							< 0·001
≥ 20: < 0·05	Ref			Ref			
≥ 20: ≥ 0·05	1·24	1·11, 1·39	< 0·001	1·14	0·99, 1·32	0·064	
< 20: < 0·05	1·82	1·57, 2·10	< 0·001	1·45	1·26, 1·68	< 0·001	
< 20: ≥ 0·05	2·03	1·76, 2·36	< 0·001	1·62	1·38, 1·91	< 0·001	

Ref, reference; PIR, poverty to income ratio.

Model 1: the crude model.

Model 2: adjusted for age, race, PIR, education, birth weight, physical activity, sedentary time, maternal smoking during pregnancy, and household smokers.

### Subgroup analysis of the combined effects of serum vitamin-D and cotinine on the risk of overweight and obesity in children

#### Gender

Serum vitamin-D < 20 ng/ml and cotinine < 0·05 ng/ml and serum vitamin-D < 20 ng/ml and cotinine ≥ 0·05 ng/ml were related to increased risk of overweight and obesity in boys. Serum vitamin-D ≥ 20 ng/ml and cotinine ≥ 0·05 ng/ml, serum vitamin-D < 20 ng/ml and cotinine < 0·05 ng/ml and serum vitamin-D < 20 ng/ml and cotinine ≥ 0·05 ng/ml were associated with elevated risk of overweight and obesity in girls (Table 4).

**Table 4.** Subgroup analysis of the combined effects of serum vitamin-D and cotinine on the risk of overweight and obesity in children (odds ratios and 95 % confidence intervals)

Variables	Gender: Boys			Gender: Girls		
	OR	95 % CI	*P*	OR	95 % CI	*P*
Serum Vitamin-D: Cotinine						
≥ 20: < 0·05		Ref			Ref	
≥ 20: ≥ 0·05	1·04	0·88, 1·23	0·642	1·29	1·03, 1·61	0·027
< 20: < 0·05	1·50	1·20, 1·87	< 0·001	1·44	1·17, 1·77	< 0·001
< 20: ≥ 0·05	1·39	1·12, 1·72	0·004	1·89	1·46, 2·44	< 0·001

Variables	Age: < 12			Age: ≥ 12		
	OR	95 % CI	*P*	OR	95 % CI	*P*
Serum Vitamin-D: Cotinine						
≥ 20: < 0·05		Ref			Ref	
≥ 20: ≥ 0·05	1·03	0·88, 1·21	0·724	1·25	0·97, 1·62	0·080
< 20: < 0·05	1·47	1·13, 1·92	0·005	1·62	1·34, 1·96	< 0·001
< 20: ≥ 0·05	1·63	1·27, 2·08	< 0·001	1·74	1·37, 2·21	< 0·001

Variables	Household smokers: Nobody			Household smokers: ≥ 1		
	OR	95 % CI	*P*	OR	95 % CI	*P*
Serum Vitamin-D: Cotinine						
≥ 20: < 0·05		Ref			Ref	
≥ 20: ≥ 0·05	1·16	1·01, 1·33	0·033	0·71	0·33, 1·49	0·356
< 20: < 0·05	1·43	1·24, 1·66	< 0·001	1·19	0·46, 3·07	0·720
< 20: ≥ 0·05	1·49	1·23, 1·79	< 0·001	1·35	0·66, 2·79	0·405

	Race: White			Race: Black			Race: Other		
Variables	OR	95 % CI	*P*	OR	95 % CI	*P*	OR	95 % CI	*P*
Serum Vitamin-D: Cotinine									
≥ 20: < 0·05	Ref			Ref			Ref		
≥ 20: ≥ 0·05	1·16	0·91, 1·47	0·226	1·16	0·87, 1·54	0·311	1·11	0·95, 1·29	0·206
< 20: < 0·05	1·22	0·76, 1·96	0·407	1·31	0·99, 1·74	0·061	1·54	1·29, 1·83	< 0·001
< 20: ≥ 0·05	2·17	1·44, 3·29	< 0·001	1·54	1·16, 2·06	0·003	1·47	1·11, 1·94	0·007

PIR, poverty to income ratio; Ref, reference.

Model 1: the crude model.

Model 2: if not stratified, adjusted for age, race, PIR, education, birth weight, physical activity, sedentary time, maternal smoking during pregnancy, and household smokers.

#### Age

Serum vitamin-D < 20 ng/ml and cotinine < 0·05 ng/ml and serum vitamin-D < 20 ng/ml and cotinine ≥ 0·05 ng/ml were related to increased risk of overweight and obesity in both participants aged < 12 years and ≥ 12 years (Table 4).

#### Household smokers

Serum vitamin-D ≥ 20 ng/ml and cotinine ≥ 0·05 ng/ml, serum vitamin-D < 20 ng/ml and cotinine < 0·05 ng/ml and serum vitamin-D < 20 ng/ml and cotinine ≥ 0·05 ng/ml were associated with elevated risk of overweight and obesity in subjects with household smokers (Table 4).

#### Race

The increased risk of overweight and obesity was observed in White or Black with serum vitamin-D < 20 ng/ml and cotinine ≥ 0·05 ng/ml. Serum vitamin-D < 20 ng/ml and cotinine < 0·05 ng/ml and serum vitamin-D < 20 ng/ml and cotinine ≥ 0·05 ng/ml were associated with elevated risk of overweight and obesity in other races (Table 4).

## Discussion

The present study evaluated the associations of serum vitamin-D level and tobacco exposure with the risk of overweight and obesity in children. In addition, the combined effects of serum vitamin-D level and tobacco exposure on the risk of overweight and obesity in children were investigated. The results revealed that serum vitamin-D < 20 ng/ml or cotinine ≥ 0·05 ng/ml was associated with increased risk of overweight and obesity in children. Moreover, we observed the combined effects of serum vitamin-D and cotinine on the risk of overweight and obesity in children. The findings might provide a reference for achieving benefits through the joint implementation of multiple risk prevention and control strategies in the future.

In a previous study, a high prevalence of serum 25(OH)D deficiency in children between 4 and 14 years old in Chile (80·4 %) was identified, and the study revealed that obese and overweight children had the highest prevalence of serum 25(OH)D deficiency^(25)^. In addition, 25-OH-D insufficiency was common in children and adolescents in Bucaramanga, Colombia, which was associated with overweight or obesity^(26)^. Akter et al. depicted that vitamin-D deficiency and Vitamin-D receptor gene polymorphisms were correlated with the risk of obesity in children^(27)^. The inverse association between serum vitamin-D levels and measures of adiposity in children was also observed^(28)^. These findings might support the results in the current study, which demonstrated the association between serum vitamin-D < 20 ng/ml and increased risk of overweight and obesity in children. On the other hand, the exposure to in utero maternal smoking and current exposure to household environmental tobacco smoke were found positively associated with childhood overweight/obesity^(29)^. Another study similarly observed a positive correlation between increasing levels of cotinine and the risk of overweight and obesity in children and adolescents^(30)^. The results of the above studies provide evidence to the findings of our study. We found that cotinine ≥ 0·05 ng/ml was associated with increased risk of overweight and obesity in children.

In prior studies, tobacco smoke exposure was reported to be an independent predictor of vitamin D deficiency in children^(31,32)^. The interaction between tobacco smoke exposure and low vitamin D on disease was not reported. In our study, we also did not identify the interaction between tobacco smoke exposure and low vitamin D on the risk of overweight and obesity in children. Therefore, we further explore the joint effects of serum vitamin-D and tobacco smoke exposure on the risk of overweight and obesity in children. We found that the combined effects of serum vitamin-D < 20 ng/ml and cotinine ≥ 0·05 ng/ml on the risk of overweight and obesity in children was identified. The result was allied with formerly published literatures. Previously, Knihtilä et al. found that cumulative tobacco smoke exposure from pregnancy to childhood was associated with dose- and duration-dependent decreases in child lung function at 6 years, and this effect was modulated by gestational vitamin D^(15)^. Co-exposure to passive smoking and vitamin D deficiency was reported to be associated with an elevated risk of spontaneous abortion^(16)^. Shen et al. found synergistic effects of secondhand smoke and vitamin-D deficiency on the prevalence of hypertension in American women of childbearing age, with more significant effects in women who were overweight or Non-Hispanic Black^(17)^. The results in our study might help achieve more than expected benefits through the joint implementation of multiple risk prevention and control strategies. Sufficient vitamin-D supplement combined with cotinine exposure prevention might be a novel direction for weight management in children.

There were some limitations in this study. First, only associations among vitamin-D, cotinine exposure and weight in children, whether there were causal relationships still needs exploration. Second, the sample of NHANES was representative of children and adolescents in the USA, and further validation is required in other countries and regions. Third, the data such as diet and lifestyle were obtained by self-report, which may have a certain recall bias. In the future, more well-designed studies concerning the associations of Vitamin-D, cotinine exposure and weight in children were required to verify the findings in the current study.

### Conclusions

The associations of serum vitamin-D level and tobacco exposure as well as their combined effects with the risk of overweight and obesity in children were evaluated in this study. The data indicated that serum vitamin-D and cotinine exposure had combined effects on the risk of overweight and obesity in children. These findings might provide an insight that the detection of serum vitamin-D levels and reduction of tobacco exposure might be important in reducing the risk of overweight and obesity in children.

## Supporting information

Lin et al. supplementary material Lin et al. supplementary material

## Supplementary material

For supplementary material/s referred to in this article, please visit https://doi.org/10.1017/S0007114524002071
