# Supplementary material for: The joint effect of vitamin-D status and tobacco exposure on overweight and obesity in children
Source: Br J Nutr. 2024 Nov 6;132(10):1386–93. doi: 10.1017/S0007114524002071 (PMC11646676; doi:10.1017/S0007114524002071)
Supplement: Lin et al. supplementary material [file S0007114524002071sup001.docx]

Supplementary Table 1 The status of missing values in this study

| Variables | n (%) |
| --- | --- |
| Education level | 374 (3.21) |
| Household smokers | 126 (1.08) |
| Sedentary time | 236 (2.03) |

Supplementary Table 2 The sensitivity analysis of data before and after missing value imputation

| Variables | After imputation (n=11636) | Before imputation (n=11636) | Statistics | *P* |
| --- | --- | --- | --- | --- |
| Education level, n (%) |  |  | χ^2^=5.866 | 0.053 |
| Below high school | 3121 (20.43) | 3027 (20.23) |  |  |
| High school | 3254 (27.16) | 3137 (27.06) |  |  |
| Above high school | 5261 (52.41) | 5098 (52.71) |  |  |
| Household smokers, n (%) |  |  | χ^2^=0.153 | 0.696 |
| 0 | 10168 (88.39) | 10061 (88.41) |  |  |
| ≥1 | 1468 (11.61) | 1449 (11.59) |  |  |
| Sedentary time |  |  | χ^2^=0.037 | 0.847 |
| <5h | 7467 (63.36) | 7311 (63.38) |  |  |
| ≥5h | 4169 (36.64) | 4089 (36.62) |  |  |

Supplementary Table 3 Univariable analysis of the potential covariates associated with the risk of overweight and obesity in children

| Variables | OR (95%CI) | *P* |
| --- | --- | --- |
| Age | 1.03 (1.02-1.05) | <0.001 |
| Gender |  |  |
| Male | Ref |  |
| Female | 1.03 (0.93-1.14) | 0.543 |
| Race |  |  |
| White | Ref |  |
| Black | 1.38 (1.21-1.58) | <0.001 |
| Other | 1.41 (1.28-1.56) | <0.001 |
| PIR |  |  |
| <1.3 | Ref |  |
| ≥1.3 | 0.76 (0.69-0.83) | <0.001 |
| Unknown | 0.95 (0.76-1.18) | 0.616 |
| Education level |  |  |
| Below high school | Ref |  |
| High school | 0.87 (0.77-0.99) | 0.039 |
| Above high school | 0.65 (0.58-0.73) | <0.001 |
| Birth weight (pounds) |  |  |
| <5.5 | Ref |  |
| ≥5.5 | 1.21 (1.01-1.46) | 0.044 |
| Unknown | 1.21 (0.98-1.50) | 0.076 |
| Physical activity |  |  |
| Not ideal physical activity | Ref |  |
| Ideal physical activity | 0.78 (0.68-0.89) | <0.001 |
| Unknown | 0.90 (0.76-1.08) | 0.271 |
| Sedentary time (hours) |  |  |
| <5 | Ref |  |
| ≥5 | 1.28 (1.16-1.41) | <0.001 |
| Maternal smoking during pregnancy |  |  |
| No | Ref |  |
| Yes | 1.36 (1.16-1.60) | <0.001 |
| Unknown | 1.03 (0.89-1.18) | 0.729 |
| Household smokers |  |  |
| Nobody | Ref |  |
| ≥1 | 1.30 (1.15-1.48) | <0.001 |
| Energy | 0.97 (0.92-1.02) | 0.187 |
| Fat | 1.00 (0.95-1.05) | 0.904 |
| Protein | 1.03 (0.98-1.08) | 0.263 |
| Carbohydrate/ Fiber | 1.00 (0.95-1.06) | 0.959 |
| Vitamin D | 1.00 (0.98-1.01) | 0.466 |

OR: odds ratio, CI: confidence interval, Ref: reference, BMI: body mass index, PIR: poverty to income ratio

Supplementary Table 4 The interaction effect of serum Vitamin-D and cotinine on the risk of overweight and obesity in children

| RERI (95%CI) | APAB (95%CI) | S (95%CI) |
| --- | --- | --- |
| 0.028 (-0.256- 0.312) | 0.017 (-0.157-0.191) | 1.047 (0.654-1.675) |

OR: odds ratio, CI: confidence interval, Ref: reference, RERI: relative excess risk due to interaction, AP: attributable proportion due to interaction, S: synergy index
